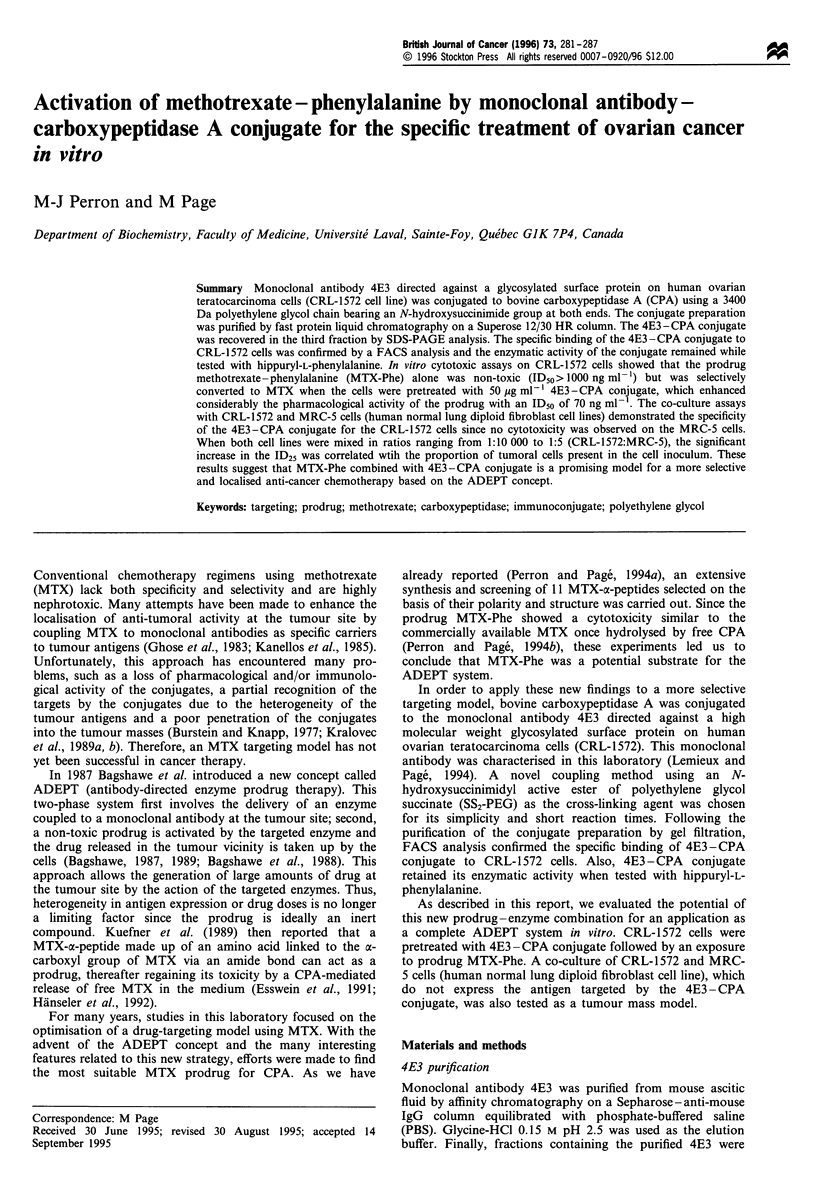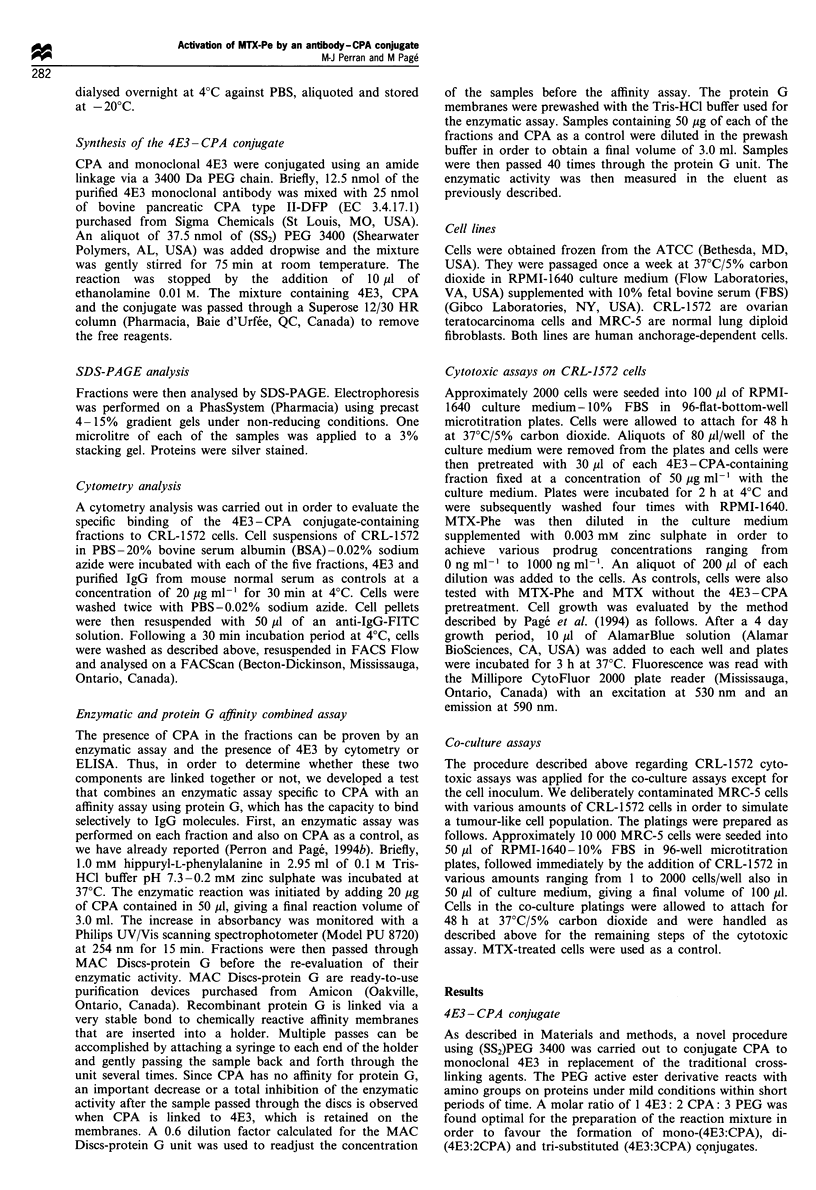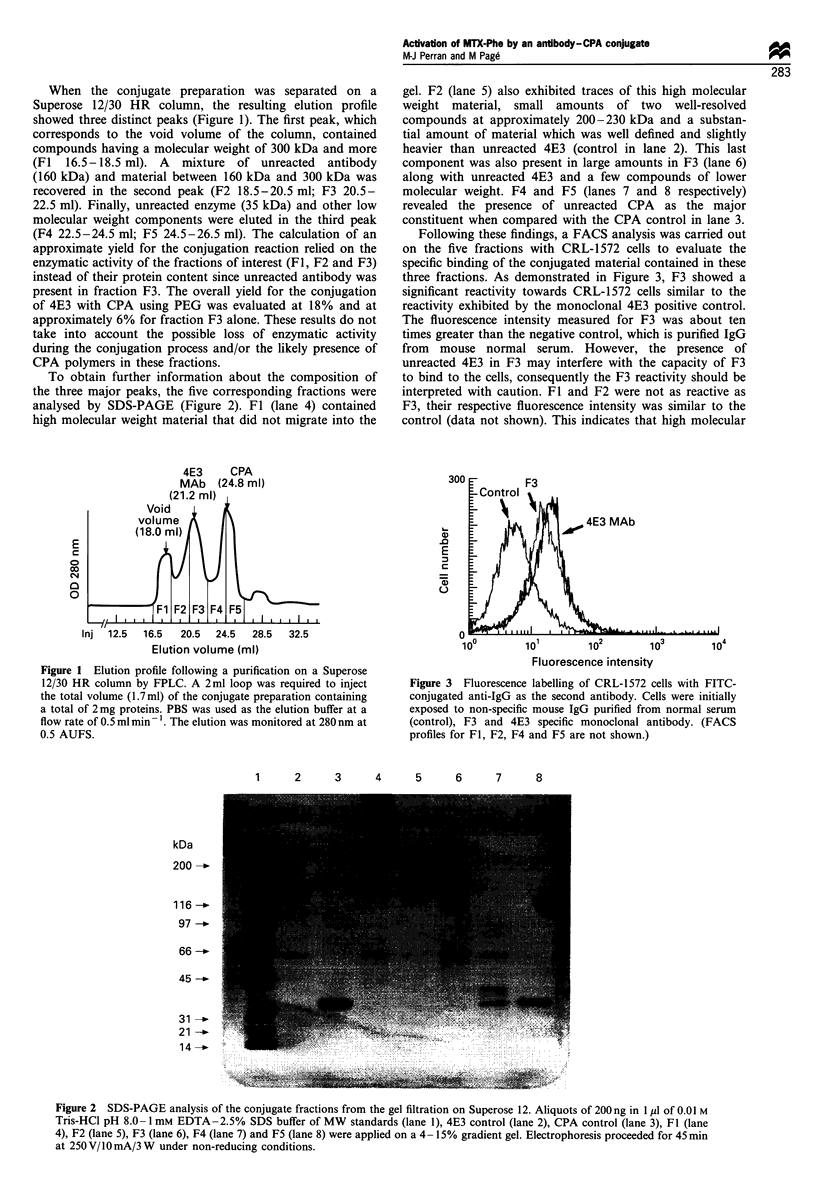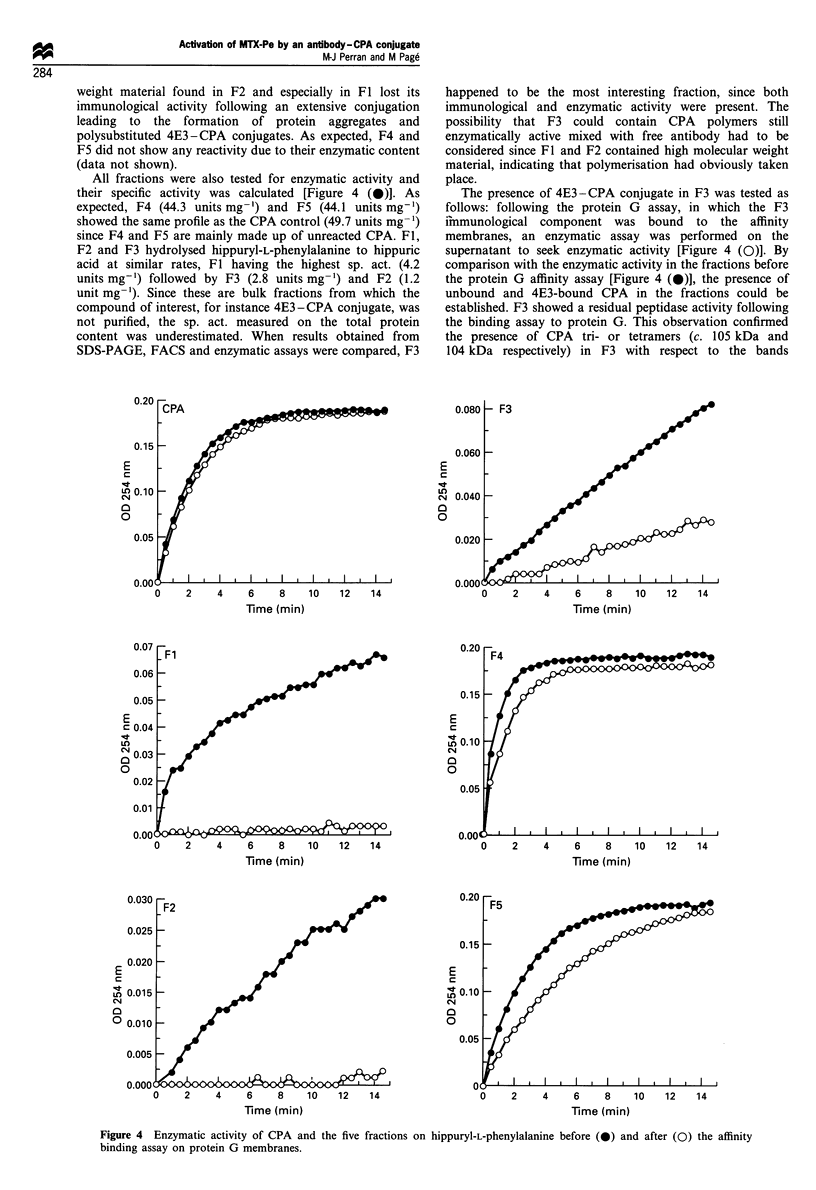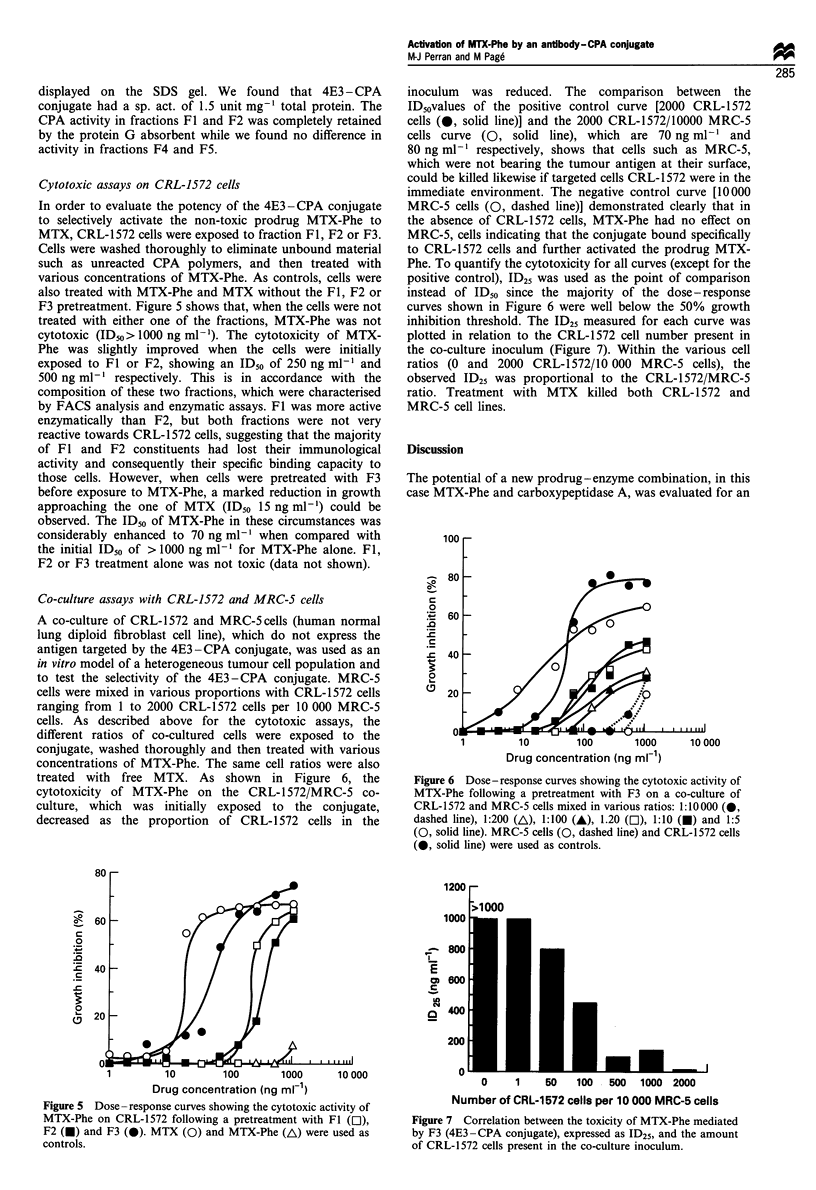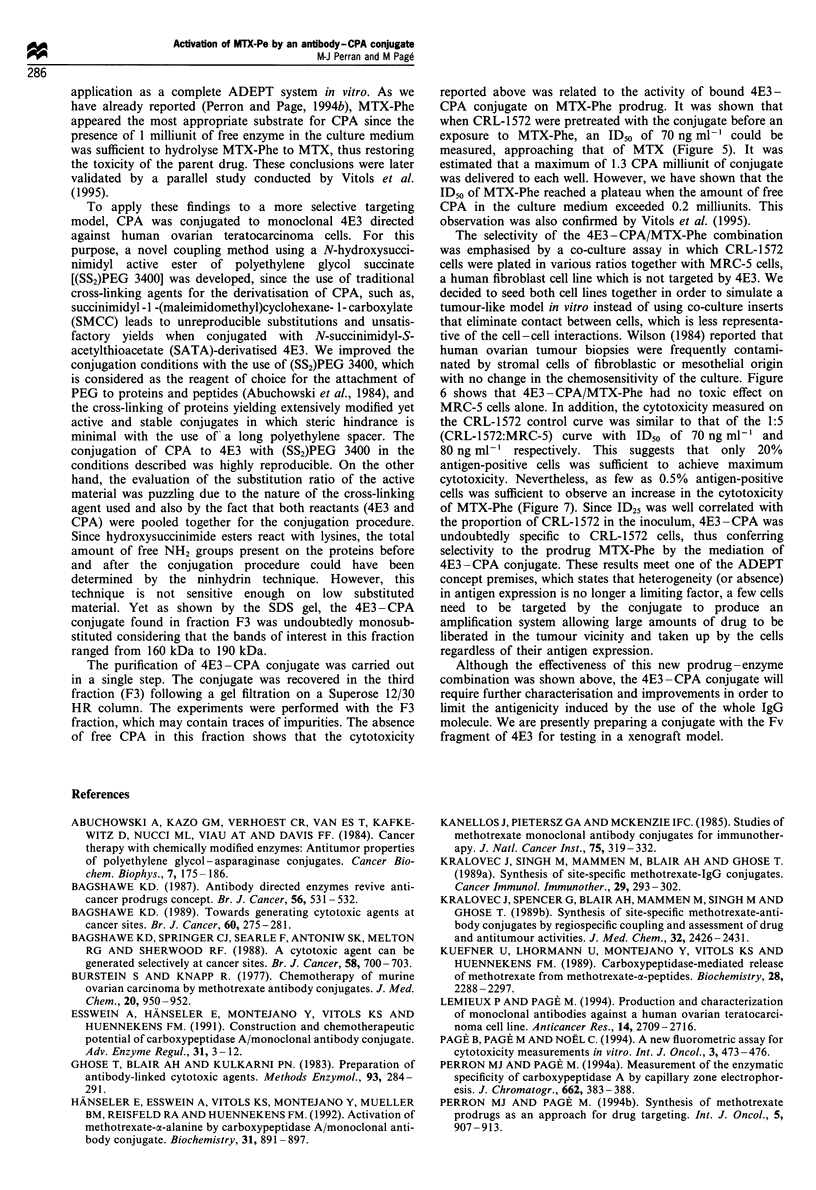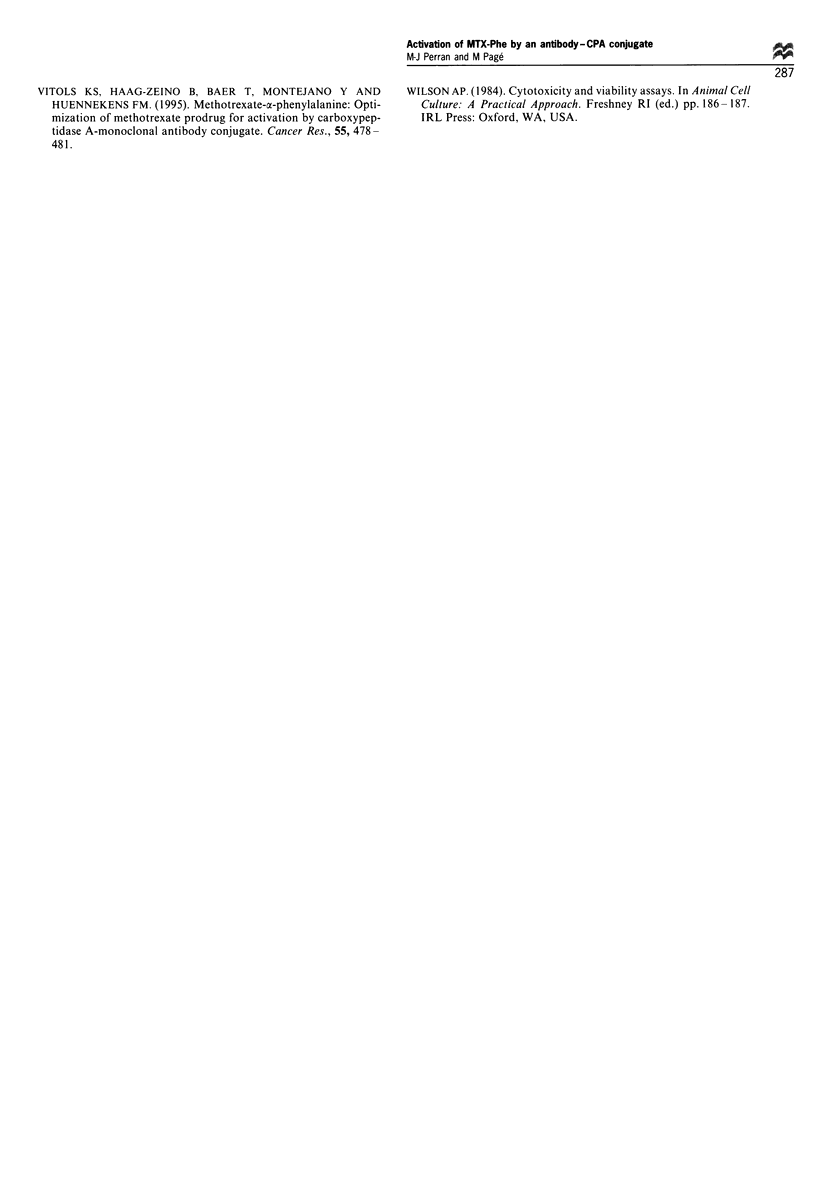# Activation of methotrexate-phenylalanine by monoclonal antibody--carboxypeptidase A conjugate for the specific treatment of ovarian cancer in vitro.

**DOI:** 10.1038/bjc.1996.50

**Published:** 1996-02

**Authors:** M. J. Perron, M. Page

**Affiliations:** Department of Biochemistry, Faculty of Medicine, Université Laval, Sainte-Foy, Québec, Canada.

## Abstract

**Images:**


British Journal of Cancer (1996) 73, 281-287
© 1996 Stockton Press All rights reserved 0007-0920/96 $12.00

### Activation of methotrexate-phenylalanine by monoclonal antibody-carboxypeptidase A conjugate for the specific treatment of ovarian cancer *in vitro*

M-J Perron and M Page

*Department of Biochemistry, Faculty of Medicine, Université Laval, Sainte-Foy, Québec G1K 7P4, Canada*

**Summary** Monoclonal antibody 4E3 directed against a glycosylated surface protein on human ovarian teratocarcinoma cells (CRL-1572 cell line) was conjugated to bovine carboxypeptidase A (CPA) using a 3400 Da polyethylene glycol chain bearing an *N*-hydroxysuccinimide group at both ends. The conjugate preparation was purified by fast protein liquid chromatography on a Superose 12/30 HR column. The 4E3-CPA conjugate was recovered in the third fraction by SDS-PAGE analysis. The specific binding of the 4E3-CPA conjugate to CRL-1572 cells was confirmed by a FACS analysis and the enzymatic activity of the conjugate remained while tested with hippuryl-L-phenylalanine. *In vitro* cytotoxic assays on CRL-1572 cells showed that the prodrug methotrexate-phenylalanine (MTX-Phe) alone was non-toxic ($ID_{50} > 1000$ ng ml$^{-1}$) but was selectively converted to MTX when the cells were pretreated with 50 $\mu$g ml$^{-1}$ 4E3-CPA conjugate, which enhanced considerably the pharmacological activity of the prodrug with an $ID_{50}$ of 70 ng ml$^{-1}$. The co-culture assays with CRL-1572 and MRC-5 cells (human normal lung diploid fibroblast cell lines) demonstrated the specificity of the 4E3-CPA conjugate for the CRL-1572 cells since no cytotoxicity was observed on the MRC-5 cells. When both cell lines were mixed in ratios ranging from 1:10 000 to 1:5 (CRL-1572:MRC-5), the significant increase in the $ID_{25}$ was correlated wtih the proportion of tumoral cells present in the cell inoculum. These results suggest that MTX-Phe combined with 4E3-CPA conjugate is a promising model for a more selective and localised anti-cancer chemotherapy based on the ADEPT concept.

**Keywords:** targeting; prodrug; methotrexate; carboxypeptidase; immunoconjugate; polyethylene glycol

Conventional chemotherapy regimens using methotrexate (MTX) lack both specificity and selectivity and are highly nephrotoxic. Many attempts have been made to enhance the localisation of anti-tumoral activity at the tumour site by coupling MTX to monoclonal antibodies as specific carriers to tumour antigens (Ghose *et al.*, 1983; Kanellos *et al.*, 1985). Unfortunately, this approach has encountered many problems, such as a loss of pharmacological and/or immunological activity of the conjugates, a partial recognition of the targets by the conjugates due to the heterogeneity of the tumour antigens and a poor penetration of the conjugates into the tumour masses (Burstein and Knapp, 1977; Kralovec *et al.*, 1989*a*, *b*). Therefore, an MTX targeting model has not yet been successful in cancer therapy.

In 1987 Bagshawe *et al.* introduced a new concept called ADEPT (antibody-directed enzyme prodrug therapy). This two-phase system first involves the delivery of an enzyme coupled to a monoclonal antibody at the tumour site; second, a non-toxic prodrug is activated by the targeted enzyme and the drug released in the tumour vicinity is taken up by the cells (Bagshawe, 1987, 1989; Bagshawe *et al.*, 1988). This approach allows the generation of large amounts of drug at the tumour site by the action of the targeted enzymes. Thus, heterogeneity in antigen expression or drug doses is no longer a limiting factor since the prodrug is ideally an inert compound. Kuefner *et al.* (1989) then reported that a MTX-α-peptide made up of an amino acid linked to the α-carboxyl group of MTX via an amide bond can act as a prodrug, thereafter regaining its toxicity by a CPA-mediated release of free MTX in the medium (Esswein *et al.*, 1991; Hänseler *et al.*, 1992).

For many years, studies in this laboratory focused on the optimisation of a drug-targeting model using MTX. With the advent of the ADEPT concept and the many interesting features related to this new strategy, efforts were made to find the most suitable MTX prodrug for CPA. As we have already reported (Perron and Pagé, 1994*a*), an extensive synthesis and screening of 11 MTX-α-peptides selected on the basis of their polarity and structure was carried out. Since the prodrug MTX-Phe showed a cytotoxicity similar to the commercially available MTX once hydrolysed by free CPA (Perron and Pagé, 1994*b*), these experiments led us to conclude that MTX-Phe was a potential substrate for the ADEPT system.

In order to apply these new findings to a more selective targeting model, bovine carboxypeptidase A was conjugated to the monoclonal antibody 4E3 directed against a high molecular weight glycosylated surface protein on human ovarian teratocarcinoma cells (CRL-1572). This monoclonal antibody was characterised in this laboratory (Lemieux and Pagé, 1994). A novel coupling method using an *N*-hydroxysuccinimidyl active ester of polyethylene glycol succinate ($SS_2$-PEG) as the cross-linking agent was chosen for its simplicity and short reaction times. Following the purification of the conjugate preparation by gel filtration, FACS analysis confirmed the specific binding of 4E3-CPA conjugate to CRL-1572 cells. Also, 4E3-CPA conjugate retained its enzymatic activity when tested with hippuryl-L-phenylalanine.

As described in this report, we evaluated the potential of this new prodrug-enzyme combination for an application as a complete ADEPT system *in vitro*. CRL-1572 cells were pretreated with 4E3-CPA conjugate followed by an exposure to prodrug MTX-Phe. A co-culture of CRL-1572 and MRC-5 cells (human normal lung diploid fibroblast cell line), which do not express the antigen targeted by the 4E3-CPA conjugate, was also tested as a tumour mass model.

### Materials and methods

### *4E3 purification*

Monoclonal antibody 4E3 was purified from mouse ascitic fluid by affinity chromatography on a Sepharose-anti-mouse IgG column equilibrated with phosphate-buffered saline (PBS). Glycine-HCl 0.15 M pH 2.5 was used as the elution buffer. Finally, fractions containing the purified 4E3 were

Correspondence: M Page
Received 30 June 1995; revised 30 August 1995; accepted 14 September 1995

dialysed overnight at 4°C against PBS, aliquoted and stored at −20°C.

*Synthesis of the 4E3-CPA conjugate*

CPA and monoclonal 4E3 were conjugated using an amide linkage via a 3400 Da PEG chain. Briefly, 12.5 nmol of the purified 4E3 monoclonal antibody was mixed with 25 nmol of bovine pancreatic CPA type II-DFP (EC 3.4.17.1) purchased from Sigma Chemicals (St Louis, MO, USA). An aliquot of 37.5 nmol of ($SS_2$) PEG 3400 (Shearwater Polymers, AL, USA) was added dropwise and the mixture was gently stirred for 75 min at room temperature. The reaction was stopped by the addition of 10 μl of ethanolamine 0.01 M. The mixture containing 4E3, CPA and the conjugate was passed through a Superose 12/30 HR column (Pharmacia, Baie d'Urfée, QC, Canada) to remove the free reagents.

*SDS-PAGE analysis*

Fractions were then analysed by SDS-PAGE. Electrophoresis was performed on a PhasSystem (Pharmacia) using precast 4-15% gradient gels under non-reducing conditions. One microlitre of each of the samples was applied to a 3% stacking gel. Proteins were silver stained.

*Cytometry analysis*

A cytometry analysis was carried out in order to evaluate the specific binding of the 4E3-CPA conjugate-containing fractions to CRL-1572 cells. Cell suspensions of CRL-1572 in PBS-20% bovine serum albumin (BSA)-0.02% sodium azide were incubated with each of the five fractions, 4E3 and purified IgG from mouse normal serum as controls at a concentration of 20 μg $ml^{-1}$ for 30 min at 4°C. Cells were washed twice with PBS-0.02% sodium azide. Cell pellets were then resuspended with 50 μl of an anti-IgG-FITC solution. Following a 30 min incubation period at 4°C, cells were washed as described above, resuspended in FACS Flow and analysed on a FACScan (Becton-Dickinson, Mississauga, Ontario, Canada).

*Enzymatic and protein G affinity combined assay*

The presence of CPA in the fractions can be proven by an enzymatic assay and the presence of 4E3 by cytometry or ELISA. Thus, in order to determine whether these two components are linked together or not, we developed a test that combines an enzymatic assay specific to CPA with an affinity assay using protein G, which has the capacity to bind selectively to IgG molecules. First, an enzymatic assay was performed on each fraction and also on CPA as a control, as we have already reported (Perron and Pagé, 1994*b*). Briefly, 1.0 mM hippuryl-L-phenylalanine in 2.95 ml of 0.1 M Tris-HCl buffer pH 7.3-0.2 mM zinc sulphate was incubated at 37°C. The enzymatic reaction was initiated by adding 20 μg of CPA contained in 50 μl, giving a final reaction volume of 3.0 ml. The increase in absorbancy was monitored with a Philips UV/Vis scanning spectrophotometer (Model PU 8720) at 254 nm for 15 min. Fractions were then passed through MAC Discs-protein G before the re-evaluation of their enzymatic activity. MAC Discs-protein G are ready-to-use purification devices purchased from Amicon (Oakville, Ontario, Canada). Recombinant protein G is linked via a very stable bond to chemically reactive affinity membranes that are inserted into a holder. Multiple passes can be accomplished by attaching a syringe to each end of the holder and gently passing the sample back and forth through the unit several times. Since CPA has no affinity for protein G, an important decrease or a total inhibition of the enzymatic activity after the sample passed through the discs is observed when CPA is linked to 4E3, which is retained on the membranes. A 0.6 dilution factor calculated for the MAC Discs-protein G unit was used to readjust the concentration of the samples before the affinity assay. The protein G membranes were prewashed with the Tris-HCl buffer used for the enzymatic assay. Samples containing 50 μg of each of the fractions and CPA as a control were diluted in the prewash buffer in order to obtain a final volume of 3.0 ml. Samples were then passed 40 times through the protein G unit. The enzymatic activity was then measured in the eluent as previously described.

*Cell lines*

Cells were obtained frozen from the ATCC (Bethesda, MD, USA). They were passaged once a week at 37°C/5% carbon dioxide in RPMI-1640 culture medium (Flow Laboratories, VA, USA) supplemented with 10% fetal bovine serum (FBS) (Gibco Laboratories, NY, USA). CRL-1572 are ovarian teratocarcinoma cells and MRC-5 are normal lung diploid fibroblasts. Both lines are human anchorage-dependent cells.

*Cytotoxic assays on CRL-1572 cells*

Approximately 2000 cells were seeded into 100 μl of RPMI-1640 culture medium-10% FBS in 96-flat-bottom-well microtitration plates. Cells were allowed to attach for 48 h at 37°C/5% carbon dioxide. Aliquots of 80 μl/well of the culture medium were removed from the plates and cells were then pretreated with 30 μl of each 4E3-CPA-containing fraction fixed at a concentration of 50 μg $ml^{-1}$ with the culture medium. Plates were incubated for 2 h at 4°C and were subsequently washed four times with RPMI-1640. MTX-Phe was then diluted in the culture medium supplemented with 0.003 mM zinc sulphate in order to achieve various prodrug concentrations ranging from 0 ng $ml^{-1}$ to 1000 ng $ml^{-1}$. An aliquot of 200 μl of each dilution was added to the cells. As controls, cells were also tested with MTX-Phe and MTX without the 4E3-CPA pretreatment. Cell growth was evaluated by the method described by Pagé *et al.* (1994) as follows. After a 4 day growth period, 10 μl of AlamarBlue solution (Alamar BioSciences, CA, USA) was added to each well and plates were incubated for 3 h at 37°C. Fluorescence was read with the Millipore CytoFluor 2000 plate reader (Mississauga, Ontario, Canada) with an excitation at 530 nm and an emission at 590 nm.

*Co-culture assays*

The procedure described above regarding CRL-1572 cytotoxic assays was applied for the co-culture assays except for the cell inoculum. We deliberately contaminated MRC-5 cells with various amounts of CRL-1572 cells in order to simulate a tumour-like cell population. The platings were prepared as follows. Approximately 10 000 MRC-5 cells were seeded into 50 μl of RPMI-1640-10% FBS in 96-well microtitration plates, followed immediately by the addition of CRL-1572 in various amounts ranging from 1 to 2000 cells/well also in 50 μl of culture medium, giving a final volume of 100 μl. Cells in the co-culture platings were allowed to attach for 48 h at 37°C/5% carbon dioxide and were handled as described above for the remaining steps of the cytotoxic assay. MTX-treated cells were used as a control.

### Results

*4E3-CPA conjugate*

As described in Materials and methods, a novel procedure using ($SS_2$)PEG 3400 was carried out to conjugate CPA to monoclonal 4E3 in replacement of the traditional cross-linking agents. The PEG active ester derivative reacts with amino groups on proteins under mild conditions within short periods of time. A molar ratio of 1 4E3 : 2 CPA : 3 PEG was found optimal for the preparation of the reaction mixture in order to favour the formation of mono-(4E3:CPA), di-(4E3:2CPA) and tri-substituted (4E3:3CPA) conjugates.

When the conjugate preparation was separated on a Superose 12/30 HR column, the resulting elution profile showed three distinct peaks (Figure 1). The first peak, which corresponds to the void volume of the column, contained compounds having a molecular weight of 300 kDa and more (Fl 16.5–18.5 ml). A mixture of unreacted antibody (160 kDa) and material between 160 kDa and 300 kDa was recovered in the second peak (F2 18.5–20.5 ml; F3 20.5–22.5 ml). Finally, unreacted enzyme (35 kDa) and other low molecular weight components were eluted in the third peak (F4 22.5–24.5 ml; F5 24.5–26.5 ml). The calculation of an approximate yield for the conjugation reaction relied on the enzymatic activity of the fractions of interest (Fl, F2 and F3) instead of their protein content since unreacted antibody was present in fraction F3. The overall yield for the conjugation of 4E3 with CPA using PEG was evaluated at 18% and at approximately 6% for fraction F3 alone. These results do not take into account the possible loss of enzymatic activity during the conjugation process and/or the likely presence of CPA polymers in these fractions.

To obtain further information about the composition of the three major peaks, the five corresponding fractions were analysed by SDS-PAGE (Figure 2). Fl (lane 4) contained high molecular weight material that did not migrate into the gel. F2 (lane 5) also exhibited traces of this high molecular weight material, small amounts of two well-resolved compounds at approximately 200–230 kDa and a substantial amount of material which was well defined and slightly heavier than unreacted 4E3 (control in lane 2). This last component was also present in large amounts in F3 (lane 6) along with unreacted 4E3 and a few compounds of lower molecular weight. F4 and F5 (lanes 7 and 8 respectively) revealed the presence of unreacted CPA as the major constituent when compared with the CPA control in lane 3.

Following these findings, a FACS analysis was carried out on the five fractions with CRL-1572 cells to evaluate the specific binding of the conjugated material contained in these three fractions. As demonstrated in Figure 3, F3 showed a significant reactivity towards CRL-1572 cells similar to the reactivity exhibited by the monoclonal 4E3 positive control. The fluorescence intensity measured for F3 was about ten times greater than the negative control, which is purified IgG from mouse normal serum. However, the presence of unreacted 4E3 in F3 may interfere with the capacity of F3 to bind to the cells, consequently the F3 reactivity should be interpreted with caution. Fl and F2 were not as reactive as F3, their respective fluorescence intensity was similar to the control (data not shown). This indicates that high molecular

**Figure 1** Elution profile following a purification on a Superose 12/30 HR column by FPLC. A 2 ml loop was required to inject the total volume (1.7 ml) of the conjugate preparation containing a total of 2 mg proteins. PBS was used as the elution buffer at a flow rate of $0.5\,\mathrm{ml\,min^{-1}}$. The elution was monitored at 280 nm at 0.5 AUFS.

**Figure 3** Fluorescence labelling of CRL-1572 cells with FITC-conjugated anti-IgG as the second antibody. Cells were initially exposed to non-specific mouse IgG purified from normal serum (control), F3 and 4E3 specific monoclonal antibody. (FACS profiles for Fl, F2, F4 and F5 are not shown.)

**Figure 2** SDS-PAGE analysis of the conjugate fractions from the gel filtration on Superose 12. Aliquots of 200 ng in 1 $\mu$l of 0.01 M Tris-HCl pH 8.0–1 mM EDTA–2.5% SDS buffer of MW standards (lane 1), 4E3 control (lane 2), CPA control (lane 3), Fl (lane 4), F2 (lane 5), F3 (lane 6), F4 (lane 7) and F5 (lane 8) were applied on a 4–15% gradient gel. Electrophoresis proceeded for 45 min at 250 V/10 mA/3 W under non-reducing conditions.

weight material found in F2 and especially in Fl lost its immunological activity following an extensive conjugation leading to the formation of protein aggregates and polysubstituted 4E3–CPA conjugates. As expected, F4 and F5 did not show any reactivity due to their enzymatic content (data not shown).

All fractions were also tested for enzymatic activity and their specific activity was calculated [Figure 4 (●)]. As expected, F4 (44.3 units $mg^{-1}$) and F5 (44.1 units $mg^{-1}$) showed the same profile as the CPA control (49.7 units $mg^{-1}$) since F4 and F5 are mainly made up of unreacted CPA. Fl, F2 and F3 hydrolysed hippuryl-L-phenylalanine to hippuric acid at similar rates, Fl having the highest sp. act. (4.2 units $mg^{-1}$) followed by F3 (2.8 units $mg^{-1}$) and F2 (1.2 unit $mg^{-1}$). Since these are bulk fractions from which the compound of interest, for instance 4E3–CPA conjugate, was not purified, the sp. act. measured on the total protein content was underestimated. When results obtained from SDS-PAGE, FACS and enzymatic assays were compared, F3 happened to be the most interesting fraction, since both immunological and enzymatic activity were present. The possibility that F3 could contain CPA polymers still enzymatically active mixed with free antibody had to be considered since Fl and F2 contained high molecular weight material, indicating that polymerisation had obviously taken place.

The presence of 4E3–CPA conjugate in F3 was tested as follows: following the protein G assay, in which the F3 immunological component was bound to the affinity membranes, an enzymatic assay was performed on the supernatant to seek enzymatic activity [Figure 4 (○)]. By comparison with the enzymatic activity in the fractions before the protein G affinity assay [Figure 4 (●)], the presence of unbound and 4E3-bound CPA in the fractions could be established. F3 showed a residual peptidase activity following the binding assay to protein G. This observation confirmed the presence of CPA tri- or tetramers (*c.* 105 kDa and 104 kDa respectively) in F3 with respect to the bands

**Figure 4** Enzymatic activity of CPA and the five fractions on hippuryl-L-phenylalanine before (●) and after (○) the affinity binding assay on protein G membranes.

displayed on the SDS gel. We found that 4E3–CPA conjugate had a sp. act. of 1.5 unit $mg^{-1}$ total protein. The CPA activity in fractions F1 and F2 was completely retained by the protein G absorbent while we found no difference in activity in fractions F4 and F5.

### *Cytotoxic assays on CRL-1572 cells*

In order to evaluate the potency of the 4E3–CPA conjugate to selectively activate the non-toxic prodrug MTX-Phe to MTX, CRL-1572 cells were exposed to fraction F1, F2 or F3. Cells were washed thoroughly to eliminate unbound material such as unreacted CPA polymers, and then treated with various concentrations of MTX-Phe. As controls, cells were also treated with MTX-Phe and MTX without the F1, F2 or F3 pretreatment. Figure 5 shows that, when the cells were not treated with either one of the fractions, MTX-Phe was not cytotoxic ($ID_{50}$ > 1000 ng $ml^{-1}$). The cytotoxicity of MTX-Phe was slightly improved when the cells were initially exposed to F1 or F2, showing an $ID_{50}$ of 250 ng $ml^{-1}$ and 500 ng $ml^{-1}$ respectively. This is in accordance with the composition of these two fractions, which were characterised by FACS analysis and enzymatic assays. F1 was more active enzymatically than F2, but both fractions were not very reactive towards CRL-1572 cells, suggesting that the majority of F1 and F2 constituents had lost their immunological activity and consequently their specific binding capacity to those cells. However, when cells were pretreated with F3 before exposure to MTX-Phe, a marked reduction in growth approaching the one of MTX ($ID_{50}$ 15 ng $ml^{-1}$) could be observed. The $ID_{50}$ of MTX-Phe in these circumstances was considerably enhanced to 70 ng $ml^{-1}$ when compared with the initial $ID_{50}$ of > 1000 ng $ml^{-1}$ for MTX-Phe alone. F1, F2 or F3 treatment alone was not toxic (data not shown).

### *Co-culture assays with CRL-1572 and MRC-5 cells*

A co-culture of CRL-1572 and MRC-5 cells (human normal lung diploid fibroblast cell line), which do not express the antigen targeted by the 4E3–CPA conjugate, was used as an *in vitro* model of a heterogeneous tumour cell population and to test the selectivity of the 4E3–CPA conjugate. MRC-5 cells were mixed in various proportions with CRL-1572 cells ranging from 1 to 2000 CRL-1572 cells per 10 000 MRC-5 cells. As described above for the cytotoxic assays, the different ratios of co-cultured cells were exposed to the conjugate, washed thoroughly and then treated with various concentrations of MTX-Phe. The same cell ratios were also treated with free MTX. As shown in Figure 6, the cytotoxicity of MTX-Phe on the CRL-1572/MRC-5 co-culture, which was initially exposed to the conjugate, decreased as the proportion of CRL-1572 cells in the inoculum was reduced. The comparison between the $ID_{50}$values of the positive control curve [2000 CRL-1572 cells (●, solid line)] and the 2000 CRL-1572/10000 MRC-5 cells curve (○, solid line), which are 70 ng $ml^{-1}$ and 80 ng $ml^{-1}$ respectively, shows that cells such as MRC-5, which were not bearing the tumour antigen at their surface, could be killed likewise if targeted cells CRL-1572 were in the immediate environment. The negative control curve [10 000 MRC-5 cells (○, dashed line)] demonstrated clearly that in the absence of CRL-1572 cells, MTX-Phe had no effect on MRC-5, cells indicating that the conjugate bound specifically to CRL-1572 cells and further activated the prodrug MTX-Phe. To quantify the cytotoxicity for all curves (except for the positive control), $ID_{25}$ was used as the point of comparison instead of $ID_{50}$ since the majority of the dose–response curves shown in Figure 6 were well below the 50% growth inhibition threshold. The $ID_{25}$ measured for each curve was plotted in relation to the CRL-1572 cell number present in the co-culture inoculum (Figure 7). Within the various cell ratios (0 and 2000 CRL-1572/10 000 MRC-5 cells), the observed $ID_{25}$ was proportional to the CRL-1572/MRC-5 ratio. Treatment with MTX killed both CRL-1572 and MRC-5 cell lines.

### Discussion

The potential of a new prodrug–enzyme combination, in this case MTX-Phe and carboxypeptidase A, was evaluated for an

**Figure 5** Dose–response curves showing the cytotoxic activity of MTX-Phe on CRL-1572 following a pretreatment with F1 (□), F2 (■) and F3 (●). MTX (○) and MTX-Phe (△) were used as controls.

**Figure 6** Dose–response curves showing the cytotoxic activity of MTX-Phe following a pretreatment with F3 on a co-culture of CRL-1572 and MRC-5 cells mixed in various ratios: 1:10 000 (●, dashed line), 1:200 (△), 1:100 (▲), 1.20 (□), 1:10 (■) and 1:5 (○, solid line). MRC-5 cells (○, dashed line) and CRL-1572 cells (●, solid line) were used as controls.

**Figure 7** Correlation between the toxicity of MTX-Phe mediated by F3 (4E3–CPA conjugate), expressed as $ID_{25}$, and the amount of CRL-1572 cells present in the co-culture inoculum.

application as a complete ADEPT system *in vitro*. As we have already reported (Perron and Page, 1994*b*), MTX-Phe appeared the most appropriate substrate for CPA since the presence of 1 milliunit of free enzyme in the culture medium was sufficient to hydrolyse MTX-Phe to MTX, thus restoring the toxicity of the parent drug. These conclusions were later validated by a parallel study conducted by Vitols *et al.* (1995).

To apply these findings to a more selective targeting model, CPA was conjugated to monoclonal 4E3 directed against human ovarian teratocarcinoma cells. For this purpose, a novel coupling method using a *N*-hydroxysuccinimidyl active ester of polyethylene glycol succinate [$(SS_2)$PEG 3400] was developed, since the use of traditional cross-linking agents for the derivatisation of CPA, such as, succinimidyl-1-(maleimidomethyl)cyclohexane-1-carboxylate (SMCC) leads to unreproducible substitutions and unsatisfactory yields when conjugated with *N*-succinimidyl-*S*-acetylthioacetate (SATA)-derivatised 4E3. We improved the conjugation conditions with the use of $(SS_2)$PEG 3400, which is considered as the reagent of choice for the attachment of PEG to proteins and peptides (Abuchowski *et al.*, 1984), and the cross-linking of proteins yielding extensively modified yet active and stable conjugates in which steric hindrance is minimal with the use of a long polyethylene spacer. The conjugation of CPA to 4E3 with $(SS_2)$PEG 3400 in the conditions described was highly reproducible. On the other hand, the evaluation of the substitution ratio of the active material was puzzling due to the nature of the cross-linking agent used and also by the fact that both reactants (4E3 and CPA) were pooled together for the conjugation procedure. Since hydroxysuccinimide esters react with lysines, the total amount of free $NH_2$ groups present on the proteins before and after the conjugation procedure could have been determined by the ninhydrin technique. However, this technique is not sensitive enough on low substituted material. Yet as shown by the SDS gel, the 4E3–CPA conjugate found in fraction F3 was undoubtedly monosubstituted considering that the bands of interest in this fraction ranged from 160 kDa to 190 kDa.

The purification of 4E3–CPA conjugate was carried out in a single step. The conjugate was recovered in the third fraction (F3) following a gel filtration on a Superose 12/30 HR column. The experiments were performed with the F3 fraction, which may contain traces of impurities. The absence of free CPA in this fraction shows that the cytotoxicity reported above was related to the activity of bound 4E3–CPA conjugate on MTX-Phe prodrug. It was shown that when CRL-1572 were pretreated with the conjugate before an exposure to MTX-Phe, an $ID_{50}$ of 70 ng ml$^{-1}$ could be measured, approaching that of MTX (Figure 5). It was estimated that a maximum of 1.3 CPA milliunit of conjugate was delivered to each well. However, we have shown that the $ID_{50}$ of MTX-Phe reached a plateau when the amount of free CPA in the culture medium exceeded 0.2 milliunits. This observation was also confirmed by Vitols *et al.* (1995).

The selectivity of the 4E3–CPA/MTX-Phe combination was emphasised by a co-culture assay in which CRL-1572 cells were plated in various ratios together with MRC-5 cells, a human fibroblast cell line which is not targeted by 4E3. We decided to seed both cell lines together in order to simulate a tumour-like model *in vitro* instead of using co-culture inserts that eliminate contact between cells, which is less representative of the cell–cell interactions. Wilson (1984) reported that human ovarian tumour biopsies were frequently contaminated by stromal cells of fibroblastic or mesothelial origin with no change in the chemosensitivity of the culture. Figure 6 shows that 4E3–CPA/MTX-Phe had no toxic effect on MRC-5 cells alone. In addition, the cytotoxicity measured on the CRL-1572 control curve was similar to that of the 1:5 (CRL-1572:MRC-5) curve with $ID_{50}$ of 70 ng ml$^{-1}$ and 80 ng ml$^{-1}$ respectively. This suggests that only 20% antigen-positive cells was sufficient to achieve maximum cytotoxicity. Nevertheless, as few as 0.5% antigen-positive cells was sufficient to observe an increase in the cytotoxicity of MTX-Phe (Figure 7). Since $ID_{25}$ was well correlated with the proportion of CRL-1572 in the inoculum, 4E3–CPA was undoubtedly specific to CRL-1572 cells, thus conferring selectivity to the prodrug MTX-Phe by the mediation of 4E3–CPA conjugate. These results meet one of the ADEPT concept premises, which states that heterogeneity (or absence) in antigen expression is no longer a limiting factor, a few cells need to be targeted by the conjugate to produce an amplification system allowing large amounts of drug to be liberated in the tumour vicinity and taken up by the cells regardless of their antigen expression.

Although the effectiveness of this new prodrug–enzyme combination was shown above, the 4E3–CPA conjugate will require further characterisation and improvements in order to limit the antigenicity induced by the use of the whole IgG molecule. We are presently preparing a conjugate with the Fv fragment of 4E3 for testing in a xenograft model.

### References

ABUCHOWSKI A, KAZO GM, VERHOEST CR, VAN ES T, KAFKEWITZ D, NUCCI ML, VIAU AT AND DAVIS FF. (1984). Cancer therapy with chemically modified enzymes: Antitumor properties of polyethylene glycol–asparaginase conjugates. *Cancer Biochem. Biophys.*, **7**, 175–186.

BAGSHAWE KD. (1987). Antibody directed enzymes revive anticancer prodrugs concept. *Br. J. Cancer*, **56**, 531–532.

BAGSHAWE KD. (1989). Towards generating cytotoxic agents at cancer sites. *Br. J. Cancer*, **60**, 275–281.

BAGSHAWE KD, SPRINGER CJ, SEARLE F, ANTONIW SK, MELTON RG AND SHERWOOD RF. (1988). A cytotoxic agent can be generated selectively at cancer sites. *Br. J. Cancer*, **58**, 700–703.

BURSTEIN S AND KNAPP R. (1977). Chemotherapy of murine ovarian carcinoma by methotrexate antibody conjugates. *J. Med. Chem.*, **20**, 950–952.

ESSWEIN A, HÄNSELER E, MONTEJANO Y, VITOLS KS AND HUENNEKENS FM. (1991). Construction and chemotherapeutic potential of carboxypeptidase A/monoclonal antibody conjugate. *Adv. Enzyme Regul.*, **31**, 3–12.

GHOSE T, BLAIR AH AND KULKARNI PN. (1983). Preparation of antibody-linked cytotoxic agents. *Methods Enzymol.*, **93**, 284–291.

HÄNSELER E, ESSWEIN A, VITOLS KS, MONTEJANO Y, MUELLER BM, REISFELD RA AND HUENNEKENS FM. (1992). Activation of methotrexate-α-alanine by carboxypeptidase A/monoclonal antibody conjugate. *Biochemistry*, **31**, 891–897.

KANELLOS J, PIETERSZ GA AND MCKENZIE IFC. (1985). Studies of methotrexate monoclonal antibody conjugates for immunotherapy. *J. Natl. Cancer Inst.*, **75**, 319–332.

KRALOVEC J, SINGH M, MAMMEN M, BLAIR AH AND GHOSE T. (1989a). Synthesis of site-specific methotrexate-IgG conjugates. *Cancer Immunol. Immunother.*, **29**, 293–302.

KRALOVEC J, SPENCER G, BLAIR AH, MAMMEN M, SINGH M AND GHOSE T. (1989b). Synthesis of site-specific methotrexate-antibody conjugates by regiospecific coupling and assessment of drug and antitumour activities. *J. Med. Chem.*, **32**, 2426–2431.

KUEFNER U, LHORMANN U, MONTEJANO Y, VITOLS KS AND HUENNEKENS FM. (1989). Carboxypeptidase-mediated release of methotrexate from methotrexate-α-peptides. *Biochemistry*, **28**, 2288–2297.

LEMIEUX P AND PAGÈ M. (1994). Production and characterization of monoclonal antibodies against a human ovarian teratocarcinoma cell line. *Anticancer Res.*, **14**, 2709–2716.

PAGÈ B, PAGÈ M AND NOËL C. (1994). A new fluorometric assay for cytotoxicity measurements *in vitro*. *Int. J. Oncol.*, **3**, 473–476.

PERRON MJ AND PAGÈ M. (1994a). Measurement of the enzymatic specificity of carboxypeptidase A by capillary zone electrophoresis. *J. Chromatogr.*, **662**, 383–388.

PERRON MJ AND PAGÈ M. (1994b). Synthesis of methotrexate prodrugs as an approach for drug targeting. *Int. J. Oncol.*, **5**, 907–913.

VITOLS KS, HAAG-ZEINO B, BAER T, MONTEJANO Y AND HUENNEKENS FM. (1995). Methotrexate-α-phenylalanine: Optimization of methotrexate prodrug for activation by carboxypeptidase A-monoclonal antibody conjugate. *Cancer Res.*, **55,** 478–481.

WILSON AP. (1984). Cytotoxicity and viability assays. In *Animal Cell Culture: A Practical Approach.* Freshney RI (ed.) pp. 186–187. IRL Press: Oxford, WA, USA.